# fMRI fluctuations within the language network are correlated with severity of hallucinatory symptoms in schizophrenia

**DOI:** 10.1038/s41537-023-00401-9

**Published:** 2023-10-30

**Authors:** Chiara Spironelli, Marco Marino, Dante Mantini, Riccardo Montalti, Alexander R. Craven, Lars Ersland, Alessandro Angrilli, Kenneth Hugdahl

**Affiliations:** 1 Department of General Psychology, University of Padova, Padova, Italy; 2 Padova Neuroscience Center, University of Padova, Padova, Italy; 3 Movement Control and Neuroplasticity Research Group, KU Leuven, Leuven, Belgium; 4 Department of Biological and Medical Psychology, University of Bergen, Bergen, Norway; 5 Department of Clinical Engineering, Haukeland University Hospital, Bergen, Norway; 6 NORMENT Center of Excellence, Haukeland University Hospital, Bergen, Norway; 7 Division of Psychiatry, Haukeland University Hospital, Bergen, Norway; 8 Department of Radiology, Haukeland University Hospital, Bergen, Norway

**Keywords:** Schizophrenia, Neural circuits

## Abstract

Although schizophrenia (SZ) represents a complex multiform psychiatric disorder, one of its most striking symptoms are auditory verbal hallucinations (AVH). While the neurophysiological origin of this pervasive symptom has been extensively studied, there is so far no consensus conclusion on the neural correlates of the vulnerability to hallucinate. With a network-based fMRI approach, following the hypothesis of altered hemispheric dominance (Crow, 1997), we expected that LN alterations might result in self-other distinction impairments in SZ patients, and lead to the distressing subjective experiences of hearing voices. We used the independent component analysis of resting-state fMRI data, to first analyze LN connectivity in three groups of participants: SZ patients with and without hallucinations (AVH/D+ and AVH/D–, respectively), and a matched healthy control (HC) group. Then, we assessed the fMRI fluctuations using additional analyses based on fractional Amplitude of Low Frequency-Fluctuations (fALFF), both at the network- and region of interest (ROI)-level. Specific LN nodes were recruited in the right hemisphere (insula and Broca homologous area) for AVH/D+ , but not for HC and AVH/D–, consistent with a left hemisphere deficit in AVH patients. The fALFF analysis at the ROI level showed a negative correlation between fALFF Slow-4 and P1 Delusions PANSS subscale and a positive correlation between the fALFF Slow-5 and P3 Hallucination PANSS subscale for AVH/D+ only. These effects were not a consequence of structural differences between groups, as morphometric analysis did not evidence any group differences. Given the role of language as an emerging property resulting from the integration of many high-level cognitive processes and the underlying cortical areas, our results suggest that LN features from fMRI connectivity and fluctuations can be a marker of neurophysiological features characterizing SZ patients depending on their vulnerability to hallucinate.

## Introduction

Schizophrenia (SZ) is one of the most challenging psychiatric disorders to diagnose. Indeed, SZ has various symptoms that result in highly heterogeneous clinical syndromes^1^ and no consistent brain alterations or clear etiopathogenetic mechanisms have been concluded^2–8^. SZ affects young adults^9,10^ and about 20 million individuals worldwide^11,12^ with a lifetime prevalence of about 0.3–0.7%^1^ regardless of the so-called SZ paradox (i.e., a constant lifetime prevalence notwithstanding patients’ lower fertility and their reduced tendency to marry and have children)^13^.

The lack of reliable biological markers for SZ diagnosis may depend on the heterogeneous nature of this disorder that, despite presenting a large variety of symptoms, often leads to the same diagnostic taxonomy, typically paranoid SZ. In this perspective, a promising approach to studying SZ traits may be to focus on specific subgroups of patients who share common clinical symptoms, rather than a singular diagnostic classification. Among all the SZ symptoms, auditory verbal hallucinations (AVH), i.e., self-other distinction impairments, which might result in the common and distressing subjective experiences of hearing voices in the absence of corresponding external auditory stimulation, are one of the most common and distressing symptoms of SZ. AVHs affect about 60%–90% of SZ patients^14^ and induce discomfort, functional impairment and behavioral alterations^15^. AVH plays a key role in Crow’s^16,17^ hypothesis on the pathogenesis of SZ, as AVH have been associated with decreased functional left hemispheric dominance, specifically affecting speech perception areas in the left temporal lobe^18^.

Building on this, several more recent studies have suggested that SZ patients suffer from alterations in hemispheric brain communication, especially within the language network (LN) in a variety of tasks^19,20^ and linguistic experimental approaches^21–25^. A growing number of studies have also sought to directly link resting-state brain function to AVH vulnerability^20,26–28^. Notably, the resting-state condition can be seen as representing a particular condition in which individuals are not engaged in external demands from the environment. Their brain activity is shown to be spontaneously and functionally organized in networks, notably the default mode network (DMN) discovered by Marcus Raichle and colleagues^29^. Although the DMN is primarily a resting-state network, it nevertheless overlaps with networks commonly modulated during engagement in active behavioral tasks^30^, including the LN^29,31^. Numerous structural and functional brain alterations have been reported in schizophrenia, and theoretical models have been suggested to explain a specific symptom/deficit from which all others are derived and considered secondary^8^. However, beyond this, most researchers in the field agree that schizophrenia is essentially a disconnection syndrome^8^. Crow linked the disconnection to language^16,17^ as this function is considered to be at the top of high-level cognitive processes, which requires the integration and connection of many cortical and subcortical brain areas. Alteration of this complex interconnected linguistic network would lead to the typical severe disorder of cognition and reality-orientation observed in schizophrenia^22,25^.

In the present study, the main goal was to investigate in SZ patients the neural correlates of the severity of the vulnerability to hallucinate in the language network. In particular, following the hypothesis of Crow, we expected that SZ might alter LN features and affect both speech perception and production. In this context, the LN represents an excellent candidate to be investigated in the domain of schizophrenia where the distinction of external self-other sources is impaired, especially when the vulnerability to hallucinatory behavior is higher. To this end, we aimed to identify neural differences between SZ patients showing higher vulnerability to hallucinatory behavior and to delusion, and the patients showing lower vulnerability. In particular, we investigate whether the vulnerability to auditory verbal hallucinations in SZ patients is associated with an altered LN, using a new approach that combines fMRI functional connectivity and spectral analysis of the oscillating functional signal. The analysis was carried out on fMRI time-series oscillations considering the fractional amplitude of low frequency fluctuations (fALFF)^32–35^. The rationale behind this innovative analysis, which is complimentary to the more typical fMRI functional connectivity, arises from the hypothesis that a similar spatial pattern in RSNs may nevertheless be associated with differences in the underlying spectral properties of the signal. According to some authors^36,37^ the fALFF analysis provides a new facet of the BOLD signal, as it measures the spectral amplitude associated with neuronal activity rather than the typical functional connectivity based on the temporal synchronization of spatially distinct brain areas. Due to this particular characteristic, the fALFF has shown to be a promising index to identify brain regions with aberrant focal functioning^37^. Such differences may depend on the study population and/or the experimental conditions under study or, in the case of clinical samples, as in the present study, on symptom severity. Therefore, following Crow’ hypothesis, we investigated the LN, as derived in a data-driven fashion through independent component analysis (ICA) in two groups of SZ patients, i.e., with higher and lower vulnerability to hallucinate (AVH/D+ and AVH/D– group, respectively), and in a group of healthy controls (HC). In this manner, we aimed to test whether AVH/D+ patients would show aberrant LN connectivity, which notably is left-lateralized, by recruiting homologous regions in the right hemisphere. In the HC and AVH/D– groups, we expected to find a typical left-lateralized LN pattern. The AVH/D– group represents an optimal control group since these patients share a common psychopathological background and comparable pharmacological treatment with AVH/D+ individuals. To test our main hypothesis, we focused on fMRI time-series data and we estimated fALFF values within the LN to investigate whether differences in the network spectral content among the groups were linked to a specific vulnerability to hallucinate. In addition, we also investigated to what extent these spectral alterations in the LN are correlated with the severity of hallucinatory symptoms. Due to the presence of AVH, we expected significant correlations between the fALFF frequency bands and P1 (delusions) - P3 (hallucinatory behavior) scores from the positive and negative syndrome scale (PANSS)^38^.

## Material and Methods

### Participants

De-identified and anonymized data from two subgroups of Norwegian SZ patients were included in the present study. The first subgroup had a predominance of positive symptoms and a score ≥ 4 on the P3 (Hallucinatory behavior) subscale of the PANSS^36,39^. The other subgroup had a predominance of negative symptoms and a score of ≤ 4 on the PANSS P3 subscale. We labelled the first subgroup AVH/D+ , and the second sub-group AVH/D–. In our study, we considered a group of SZ patients, which was then divided into two subgroups according to their vulnerability to hallucinate. The patients in the two subgroups were mutually exclusive such that if a patient obtained a P3 PANSS score of 4, we considered the severity of P1 (Delusions) item as critical to assign he/she either to the AVH/D+ (P1 score ≥ 4) or to the AVH/D– (P1 score ≤ 3) subgroup, so that there were no mixed-scoring patients. On the one hand, as main criterion to divide the SZ patient population, we first considered the P3 scores as a measure of their vulnerability to hallucinate. On the other hand, the second criterion was chosen to take into account not only abnormalities in speech perception, as assessed by the P3 subscale, but also speech production, as assessed by the P1 subscale. This is the reason why the subgrouping of the SZ patients was guided by both the P1 and P3 subscales. Indeed, for scores ≥ 4, the symptoms of both delusions (P1 subscale) and hallucinatory behavior (P3 subscale) interfere with thinking, social relations, or behavior. Therefore, to take into account not only the vulnerability to hallucinate, but also to delusions, i.e., a constellation of unfounded, unrealistic, and idiosyncratic beliefs, which are assessed considering individuals’ speech production, we also contemplated the P1 scores. Seventeen SZ patients for each subgroup satisfied the criteria, showing similar age, educational levels and gender distribution (average group means ± Standard Deviations, SD, in Table 1).

**Table 1:** Socio-demographical characteristics and medical treatment of clinical and control groups.

	Group			Statistics
	AVH/D+ (n = 17)	AVH/D–(n = 17)	HC (n = 17)	
**Age**	25.94 ± 8.56	28.38 ± 11.80	28.12 ± 7.36	*F*_2,48_ = 0.34, *n.s*.
**Gender**	6 F; 11 M	4 F; 13 M	5 F; 12 M	all χ^2^ < 1.0, *n.s*.
**Handedness**	12 R; 3 L; 1 unknown	16 R; 1 unknown	15 R; 2 L	all χ^2^ < 0.40, *n.s*.
**Illness duration**	3.91 ± 4.72	6.72 ± 11.17	–	*t*_*32*_ = -0.95*, n.s*.
**DDD**	0.50 ± 0.34	0.96 ± 0.54	–	*t*_*26*_ = −2.66,*p* = 0.01^a^
**PANSS**				
*P3*	*4.71* ± *0.59*	*2.06* ± *1.20*		*t*_*32*_ = *8.18, p* < *0.001*
*P1*	*4.53* ± *0.80*	*3.06* ± *1.20*		*t*_*32*_ = *4.21, p* < *0.001*
***Mean Positive (P1*** **+** ***P3)***	*4.62* ± *0.45*	*2.56* ± *0.86*		*t*_*32*_ = *8.71, p* < *0.001*

Mean ± Standard Deviations (SD).

Note: AVH/D+ = SZ patient with Auditory Verbal Hallucination; AVH/D– = SZ patient without Auditory Verbal Hallucination; HC = Healthy controls; DDD = Defined Daily Doses; PANSS = Positive and Negative Syndrome Scale; P3 = Hallucinations subscale; P1 = Delusions subscale. Data from the same patients and controls were used in a previous publication (Marino et al., 2022) based on different research questions and analysis.

^a^ Pharmacological data from some SZ patients (i.e., 2 AVH/D+ and 4 AVH/D–) were not available.

All patients were on second-generation antipsychotics, primarily aripiprazole, amisulpride, or olanzapine, and a few were also prescribed clozapine, quetiapine or risperidone (see Defined Daily Doses, DDD, in Table 1). The patient subgroups were compared with an age-matched control group of 17 healthy individuals. Transfer of data to University of Padua was approved by the Regional Committee for Medical Research Ethics in Western Norway (REK-Vest, #04052020-6822).

### MRI data acquisition

MR data were acquired at the Haukeland University Hospital in Bergen, Norway on a 3 T GE Discovery 750 scanner. Magnetic resonance imaging included fMRI resting state data collection during a 5.33-min eyes-closed scan. One hundred sixty whole brain volumes were acquired, with 30 slices with a 0.5 mm gap (voxel size 1.72×1.72×3 mm^3^) with the following parameters: TR = 2000 ms, TE = 30 ms, Flip Angle (FA) = 90°, and Field of View (FOV) = 220 mm. In addition, a structural T1-weighted image was acquired (7.42 min) using a 3D Spoiled Gradient-Recalled Echo (SPGR) sequence with the following parameters: TR = 7.78 ms, TE = 2.94 ms, FA = 14°, and FOV = 256 mm, with isotropic voxel size of 1 mm^3^. During all scans, participants were asked to simply stay motionless, awake and relaxed with their eyes closed; no visual or auditory stimuli were presented at any time during functional scanning. None of the subjects in the study moved, fell asleep (The patients were watched by the responsible MR-technician through a window into the scanner chamber, although not overtly monitored. It is unlikely that the patients fell asleep due to the loud background noise caused by the scanner, and no patient was found sleeping when the scanning session was over.), or reported anxiety or other particular discomfort during scanning. We cannot exclude that some patients could have experienced hallucinatory episodes. The patients were a sub-group of patients selected from a previous study^40^ where a button-press procedure was used to indicate the presence of hallucinatory episodes during scanning, and none had indicated episode presence with a button-press response. It is still possible that they experienced an episode but not indicated it with a button-press response or experienced an episode during one of the other imaging sequences.

### Clinical Assessment

The PANSS was used to quantify patients’ clinical symptoms. Due to its relevance for the study hypothesis, the severity of SZ patients’ vulnerability to hallucinate was assessed with the PANSS P3 subscale^38^. The P3 item assesses hallucinations in different modalities with a particular focus on auditory hallucinations and hearing voices, since these are the most common type of hallucination in psychotic patients. Therefore, this item provides a reasonable measure of hallucination severity in general, but the interview questions ascribed particular emphasis to AVH, that were evaluated to a greater extent than the other sensory modalities^39,40^. Together with P3, we also considered the PANSS P1 subscale, as it assesses the severity of patients’ delusional thoughts. All PANSS raters were trained and certified, and satisfactory inter-rater reliability was documented. For all patients, PANSS data were collected on the day of the fMRI scanning.

### MRI data processing

Processing of MRI data was carried out using built-in MATLAB (MathWorks, Natick, MA, United States) functions and the Statistical Parametric Mapping 12 (SPM12) software. Consistent with Marino et al. (2022)^28^, to analyze fMRI data, we used a standard preprocessing pipeline based on SPM12, including spatial alignment to structural MRI, motion correction, bias field correction, spatial smoothing (6 mm FWHM), and co-registration to standard space^28,31,41^. Then, we performed functional connectivity analysis using spatial Independent Component Analysis (ICA), which allows decomposing the fMRI data into brain patterns starting from the spatial covariance of the measured signals^42^. This led to the extraction of the spatial pattern and time-course of the Language Network (LN) at the single-subject level. The whole procedure is data-driven and was applied separately for each subject. This pipeline has been previously used in Marino et al., (2021, 2022)^28,35^ and is described in detail in the Supplementary Materials.

### Testing for between-group differences in the LN spatial map

Starting from the single-subject level LN spatial map, we derived the LN group-level correlation map by performing a one-sample t-test, using a mass-univariate analysis. According to this approach, each voxel displayed as significant in the resulting map indicates that there is a significant correlation at the group level. We corrected the significance level for multiple comparisons (for multiple voxels involved in the analysis) between single-subject t-score correlation maps using the Benjamini-Hochberg false discovery rate (BH-FDR) procedure^43^. This procedure does not make any assumptions about sample dependency. The significance threshold for the LN group-level correlation map derived from the fMRI data was set to *p* < 0.05. This was performed separately for each group to visualize the average LN functional connectivity pattern both for the HC and the SZ patients, i.e., AVH/D+ and AVH/D– groups separately. Focusing on the LN, we then performed the between-subject comparison between the HC and the SZ groups by using a two-sample t-test for the individual LN maps belonging to each group to detect regional differences in the LN map among the three groups. More specifically, group differences were tested for the HC and AVH/D–, HC and AVH/D+ , and AVH/D+ and AVH/D– contrasts. In particular, we considered the AVH/D– group as an optimal control group for the comparison with the group with higher vulnerability to hallucinatory behavior and to delusion, i.e., the AVH/D+ group, since the AVH/D– patients share a common psychopathological background and underwent the same pharmacological treatment as the AVH/D+ patients. In this way, the differences between the SZ subgroups could only be ascribable to the severity of the vulnerability, as also the definition of the daily doses (DDD) was included as covariate. This covariate was included for both the group and the between-group analyses involving the two patients’ groups. The significance threshold for these between-group comparisons was set to *p* < 0.05, BH-FDR corrected.

### Fractional amplitude of low frequency-fluctuations (fALFF)

Starting from the individual time-series associated with the LN, to estimate the fALFF, the frequency spectrum was computed using the Fast Fourier Transform (FFT) function. The fALFF was computed for the whole detectable frequency range, which was subdivided into four separate bands: slow-5 (0.01–0.027 Hz), slow-4 (0.027–0.073 Hz), slow-3 (0.073–0.198 Hz) and slow-2 (0.198– 0.25 Hz). This band separation was first suggested by Zou et al., 2008^33^ to provide a better discrimination compared to the canonical fALFF, which is computed in a narrower frequency range 0.01-0.1 Hz. Concerning the application of the fALFF on a network time-series, this has been first introduced by Esposito et al.^34^, in which altered modulations in the somatomotor network were associated with the administration of levodopa in Parkinson’s disease patient. To limit the effect of individual confound, the fALFF values in the four frequency bands were normalized with respect to the canonical fALFF^32,34^.

### Correlation of fALFF with PANSS scores

To identify the hypothesized relationships in SZ patients between neuroimaging measurements and the PANSS subscales, we performed Spearman correlation analyses between the normalized fALFF frequency band values and the P1 (Delusions) and P3 (Hallucinations) sub-scale scores.

## Results

### Socio-demographical and clinical data

No significant socio-demographical differences between HC and the SZ patient groups were found (all *t*-values and χ^2^ values < 1.0; group means ± SD in Table 1). Considering SZ sub-groups, the AVH/D– compared with AVH/D+ patients had higher dosages of second-generation antipsychotics, as revealed by significantly higher DDD values (Table 1). Furthermore, the two selected PANSS subscales revealed significant differences (all *t* tests > 4.20, *p* < 0.001), P1 and P3 scores being higher in AVH/D+ group than AVH/D–.

### ICA spatial maps for the language network (LN)

**Fig. 1:**
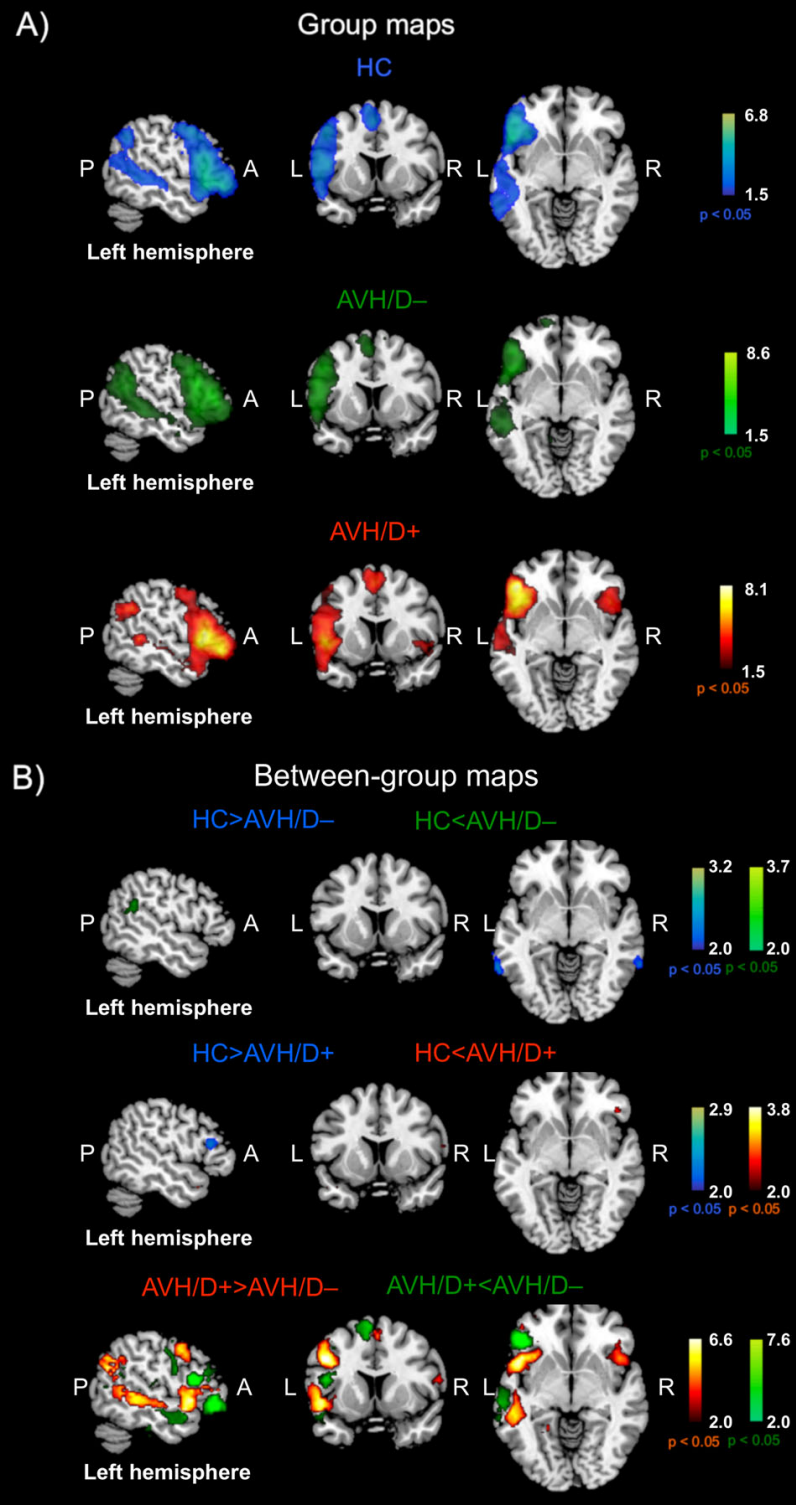
LN group maps in HC, AVH/D– and AVH/D+ patients, and between-group statistical maps. Panel **A**: Random-effects group-level maps for the LN in HC (top row, blue color scale), AVH/D– patients (middle row, green color scale), and AVH/D+ patients (bottom row, red color scale). Panel **B**: Random-effects group-level t-map for the difference between HC and AVH/D– patients (blue/green color scales depending on the group contrast), between HC and AVH/D+ patients (blue/red color scales depending on the group contrast), and between AVH/D+ and AVH/D– patients (red/green color scales depending on the group contrast), including DDD as covariate. The reported *p*-values (*p* < 0.05) were corrected for the FDR both for the random-effects group-level t-maps of each group and for the random-effects group-level t-maps of their comparison. The P, A, L and R labels stays for posterior, anterior, left and right, respectively.

Figure 1 shows the random-effects group-level maps of the LN for HC (panel A, top row, blue color scale), AVH/D– patients (panel A, middle row, green color scale) and AVH/D+ patients (panel A, bottom row, red color scale), as well as the random-effects group-level t-map for the comparison between HC and AVH/D– patients (panel B, top row, blue/green color scales), HC *vs*. AVH/D+ patients (panel B, middle row, blue/red color scales), and AVH/D+ *vs*. AVH/D– subgroups (panel B, bottom row, red/green color scales). Notably, due to significant differences in the amount of patients’ antipsychotic drugs, in the AVH/D+ and AVH/D– comparison we included the DDD as covariate.

Consistent with previous studies^31,42^ the LN primarily included brain regions in the left hemisphere, i.e., Broca’s area/frontal operculum (BA 44-45), insula (BA 13), premotor and supplementary motor areas (BA6), angular gyrus (BA 39), superior and middle temporal gyrus (BA 21-22). In addition, for the AVH/D+ group only, the LN included an atypical extra region located in the right hemisphere (Fig. 1, panel A, bottom row) on the homologous of Broca’s area/frontal operculum (BA 44) and anterior insula (BA 13).

For the HC versus AVH/D+ between-group comparison, patients had a slightly (but statistically significant) greater connectivity in the right opercular part of the inferior frontal gyrus (BA 47; MNI coordinates: 47, 32, -5) and decreased connectivity in the left triangular part of the inferior frontal gyrus (BA 45; MNI coordinates: -51, 24, 17) (Fig. 1B, middle row, red and blue color scale, for AVH/D+ > HC and AVH/D+ < HC contrasts, respectively). Notably, these regions, i.e., the right opercular part of the inferior frontal gyrus and the left triangular part of the inferior frontal gyrus, also showed a significantly greater connectivity for the comparison between AVH/D+ versus AVH/D– groups (BA 47: MNI coordinates: 43, 16, -10, and BA 45; MNI coordinates: -49, 17, 39) (Fig. 1B, bottom row, red and green color scale, for AVH/D+ > AVH/D– and AVH/D+ < AVH/D– contrasts, respectively). More specifically, these contrasts were characterized by other clusters in the right insula (BA 13; MNI coordinates: 43, 16,-10), in the left insula (BA 13; MNI coordinates: -42, 6, -12), and in the right pre-motor area (BA 6; MNI coordinates: 57, 5, 15) for the AVH/D+ > AVH/D– contrast, and in the left angular gyrus (BA 39; MNI coordinates: -54, -58, 17) and in the left dorsolateral prefrontal cortex (BA 46; MNI coordinates -51, 28, 19) for the AVH/D+ < AVH/D– contrast.

### fALFF analyses in the Language Network

**Fig. 2:**
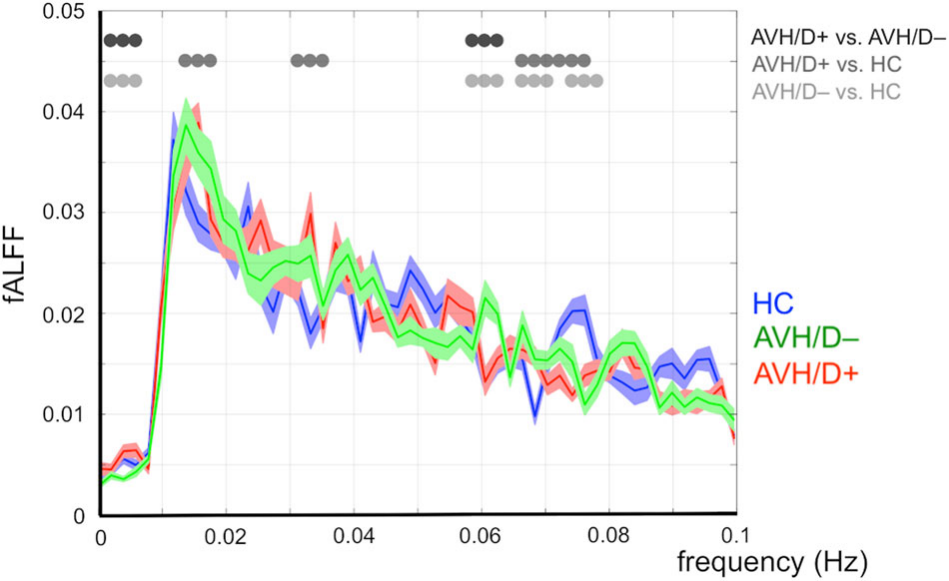
Normalized fALFF analysis carried out on LN time series in AVH/D+ patients, AVH/D– patients and HC (red, green, and blue lines, respectively). Significant differences between the AVH/D+ and AVH/D– groups, the HC and AVH/D+ groups, and the HC and AVH/D– groups, are highlighted by black, dark-gray, and light-gray dots, respectively.

Considering the normalized fALFF analysis computed from the LN time series, AVH/D+ patients showed significantly lower slow fluctuation amplitudes compared to the HC group in the frequency range between 0.012 and 0.018 Hz (all *t* values < -2.16, all *p*s < 0.04), and significantly higher amplitudes in the 0.03 and 0.036 Hz frequency range (all *t* values > 3.6, all *p*s < 0.01) (Fig. 2).

The comparison of the AVH/D– with the HC group showed significant differences for slow fluctuation amplitudes almost continuously between 0.06 and 0.08 Hz (all *t* values < -2.2, all *p*s < 0.03 for lower AVH/D– values and all *t* values > 2.3, all *p*s < 0.02 for higher AVH/D– values), whereas the AVH/D+ and AVH/D– subgroups showed significant differences at around 0.06 Hz (all *t* values > 2.03, all *p*s < 0.04) (Fig. 2).

With respect to possible associations with PANSS, we found significant positive correlations between the P1 (Delusions) subscale and Slow-4 only for the AVH/D– group. This means that the higher the amplitude of the normalized fALFF Slow-4 analysis computed from the LN time series, the greater the severity of patients’ delusions.

### Post hoc fALFF and structural MRI analyses in regions of interest

**Fig. 3:**
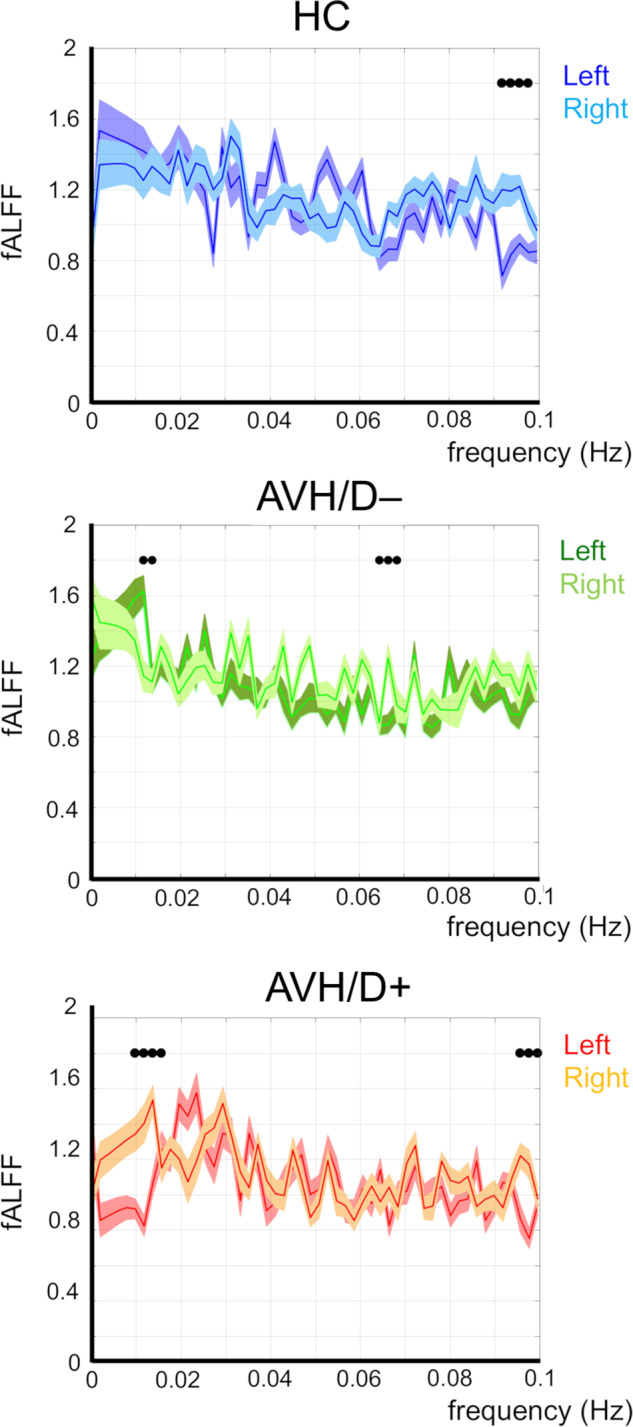
Normalized fALFF analysis carried out on left and right ROIs time series (MNI coordinates: -46, -30, 3 and 46, 30, 3, respectively) in HC, AVH/D– and AVH/D+ patients (blue/light-blue, green/light-green and red/orange lines, respectively). Significant differences (*p* < 0.05) between left and right ROIs are highlighted by black dots.

Starting from statistical t-maps showing between-group differences between AVH/D+ and HC, and between AVH/D+ and AVH/D–, we decided to carry out two additional analyses focused on AVH/D+ patients’ in a LN region that is usually not recruited by the LN due to a contralateral inhibition mechanism that ensures left hemisphere dominance for language, i.e., the right frontal operculum (homologous of Broca’s area, BA 44). Indeed, according to Crow’s hypothesis referred to in the Introduction, reduced left hemisphere language dominance represents a risk factor for SZ. As the most relevant connectivity differences between the AVH/D+ group and the other two groups were detected in the proximity of the right frontal operculum, we set a region of interest (ROI), including the Broca area and the insula in the right hemisphere, defined as a 6 mm radius sphere (BA 45; MNI coordinates: 46, 30, 3), as well as the contralateral ROI within the LN (BA 45; MNI coordinates: -46, 30, 3). For each ROI, and separately for each subject, the fALFF was calculated within the defined ROI and then normalized to limit the effect of individual confound. The normalization was performed by using the mean of the whole-brain fALFF computed across the whole detectable frequency range^44,45^. More in detail, the ROI-specific fALFF was calculated on a single representative signal of the ROI that was computed, for each ROI, by applying principal component analysis (PCA) on all the fMRI time courses from all voxels included in the spherical ROI^46,47^. By considering the first principal component resulting from this PCA, we achieved a representative activity of the whole ROI. Figure 3 shows the fALFF results for the band-analyses carried out on these ROIs, for the HC (top panel), AVH/D– (middle panel) and AVH/D+ groups (bottom panel) for left and right ROIs, respectively.

Interestingly, HC showed similar fALFF amplitude in both ROIs, apart from the higher frequency, at around 0.1 Hz, where the right ROI showed greater amplitudes than the left one (all *t* values > 2.3, all *p*s < 0.03) (Fig. 3, top panel). On the contrary, the AVH/D– group revealed greater fALFF amplitude in slow frequencies, at around 0.015 Hz (all *t* values > 2.2, all *p*s < 0.04) in the Left *vs*. Right ROI, and the opposite pattern (i.e., right > left ROI) at around 0.065 Hz (all *t* values > 2.3, all *p*s < 0.03). Notably, AVH/D+ patients exhibited significant greater fALFF amplitude in right *vs*. left ROIs at around 0.01 and 0.1 Hz (all *t* values > 2.17, all *p*s < 0.02).

**Fig. 4:**
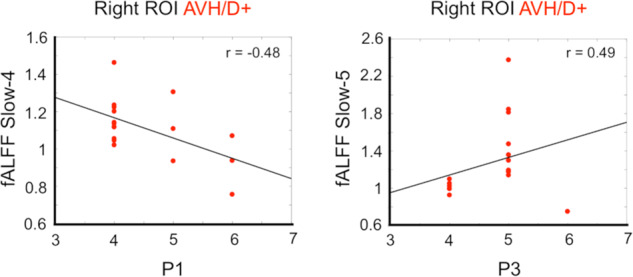
Significant Spearman correlations (*p* < 0.05) between normalized fALFF bands (ROI analysis) and the PANSS, considering the AVH/D+ group only. Significant correlations were found for Slow-4 and Slow-5 from the Right ROI with P1 (left panel) and P3 (right panel), respectively.

Considering associations with PANSS symptoms, we found that P1 scores showed significant negative correlation with Slow-4 derived from the right ROI of the AVH/D+ group (*p* < 0.04). Furthermore, P3 scores showed significant, but positive, correlation with Slow-5 from the same ROI of the same group (*p* < 0.04) (Fig. 4).

Finally, to provide a more fine-grained analysis of possible factors underlying the functional differences in ROI fALFF activity, we carried out an additional analysis at the structural level, to test whether the observed effects in the functional domain were a consequence of an underlying anatomical aberration^48^. This analysis was conducted on each individual structural MRI using the Computational Anatomy Toolbox (CAT) extension to SPM12 (http://www.neuro.uni-jena.de/cat/)^49^, which covers diverse morphometric methods. In particular, we extracted the voxel-based morphometry (VBM) to conduct a voxel-wise estimation of the grey matter volume^50^. We found no significant anatomical differences in the comparison between left and right ROIs of each patients’ group. Results revealed that compared with HC, no structural differences in left and right ROIs appeared neither in the AVH/D– nor in AVH/D+ groups (all *t* values < 1.47, all *p*s > 0.15). Furthermore, the direct comparison between AVH/D– and AVH/D+ revealed no structural differences in left and right ROIs (all *t* values < 0.57, all *p*s > 0.57; complete statistics in Table S1, in the Supplementary materials).

## Discussion

Investigations oriented towards specific SZ traits, e.g., subgroups of patients who share common clinical symptoms, rather than the same diagnostic classification, may contribute to a better understanding of SZ etiopathogenesis despite its heterogeneous nature. In our study, we expected that an increased vulnerability to hallucinate would be related to alterations in the language network (LN). Indeed, several studies have suggested that auditory verbal hallucinations are associated with decreased functional left hemispheric dominance especially in fronto-temporal brain regions specialized in the capacity to process speech, to understand its content, and to deal with complex communications and social interactions^16,17^. Notably, it has been demonstrated that SZ patients suffer from altered hemispheric communication, especially within the LN, with a variety of tasks^19,20^ and experimental approaches^21–25^. Combining resting state functional connectivity and spectral analyses, we investigated the LN in a group of healthy controls (HC), and two groups of SZ patients, i.e., AVH/D+ and AVH/D– groups, which differed for their vulnerability to hallucinate.

Firstly, within each group, we identified the relevant regions of the LN, including the recruitment of left Broca’s area/frontal operculum (BA 44-45), insula (BA 13), premotor and supplementary motor areas (BA6), angular gyrus (BA 39), superior and middle temporal gyrus (BA 21-22). As expected, in line with Crow’s hypothesis^16,17^ concerning the inverted frontal symmetry in hallucinated psychosis, AVH/D+ patients showed the recruitment of additional regions outside the LN, i.e., the homologous of Broca’s area/frontal operculum (BA 44) and the anterior right insula (BA 13). Statistical analyses confirmed these spatial LN differences showing a small, but significantly higher connectivity in the right opercular part of the inferior frontal gyrus (BA 47) of AVH/D+ patients when compared to HC. Notably, this contralateral recruitment occurred together with lower connectivity in the left triangular part of the inferior frontal gyrus (BA 45) in AVH/D+ *vs*. HC groups. These regions showed altered connectivity also in the between-patients comparison, with greater connectivity evidenced in AVH/D+ patients’ right BAs 47, 45, 13 and 6, and in the left BA 13.

From an anatomical point of view, the right frontal operculum/insula represent a critical hub, as it is located near and connected with a variety of brain areas involved in complex cognitive processes^51,52^. From a functional point of view, the insula plays a key role in a wide range of neural processes, including emotional response^53^, and language, speech, auditory, sensory-motor, decision-making, salience and attentional processing^54–56^. The insula, being involved in the integration of (external) sensory input with internal emotional processing within the limbic system, contributes to the interoception, i.e., the ability to be aware of our internal bodily state. This process represents the basis of people ability to perceive themselves as distinct from the surrounding environment, allowing individuals to be aware of themselves, and finally to distinguish the image of “self” from “not oneself”^57–59^. Focusing on language, the insula is located in the center of (and is connected with) language areas: the anterior portion extends to and interfaces with Broca’s area, whereas the posterior portion targets Wernicke’s area. Because of its relevant location, it is reasonable that insula may play a significant role in language^60^. Furthermore, an important corpus of clinical research shows that it is frequently involved in perisylvian aphasia syndromes, i.e., Broca aphasia, conduction aphasia and Wernicke aphasia^60–62^, in turn linked to alterations in temporal regions belonging to the language network. Notably, we found between-group differences also in these regions, strengthening the close relationship between insula and brain areas linked to language perception and processing. Dronkers (1996) also reported that the left precentral gyrus of the insula is involved in motor planning of speech^63^. Contemporary neuroimaging studies further support to the evidence of a significant involvement of insula in language. Activation has been demonstrated in a variety of linguistic tasks^55,64–69^.

Because of the role it plays, several studies have investigated the relationship between structural/functional abnormalities in the insula and the presence of AVH. According to some authors^70,71^, the inability to discriminate an internal sensory experience from an externally perceived one appears to mark the hallucinatory phenomenon^59^. Thus, the insula dysfunction and its functional disconnection could result in a bias leading to the misinterpretation of self-other sources, and may therefore contribute to the mechanism underlying the hallucinatory phenomena in SZ patients. Structural insula abnormalities were consistently found in schizophrenia^52^, which in turn relate to hallucinations^48^.

Secondly, we also focused on the fMRI low-frequency fluctuations computed within the LN, by applying the fractional amplitude of low frequency-fluctuations (fALFF)^28,32–35,46^, to investigate whether the spectral power profile presented differences among groups, and whether these differences were linked to the severity of hallucinatory behavior. Correlation analysis with PANSS scores showed significant associations between the PANSS subscale P1, i.e., delusions, and LN fALFF (Slow-4) only for the AVH/D– group, suggesting a relationship between greater amplitudes of LN fALFF power (in the left hemisphere, due to the typical lateralization of this network) and the severity of delusional symptoms. In other words, fALFF alterations in the LN which presents a large representation in the left hemisphere may contribute to delusional thought severity in non-hallucinated SZ patients. However, when extending the investigation to the *post hoc* ROI analysis (centered on BA 45; MNI coordinates: -46/46, 30, 3), we found significant correlations with positive symptom scores (PANSS subscales P1 and P3, hallucinatory behavior) in AVH/D+ patients only. This suggests a relationship between greater amplitudes of fALFF power spectrum in the right ROI (BA 45) and the severity of these clinical signs. Our results revealed an inverted trend between clinical symptoms and spectral power in the triangular part of the right inferior frontal gyrus. In particular, increased fALFF power was associated with greater severity of hallucination, whereas decreased fALFF power was linked to greater delusional thoughts (Fig. 4), which could be linked to previous findings of brain hyper-activity associated with AVH^72^. As above-mentioned, the P3 item assesses hallucinations in different modalities, and for the purposes of the present study, the PANSS interviewer ascribed particular emphasis to AVH and hearing voices. Our results revealed a clear pattern of abnormal connectivity specific for AVH/D+ patients: for this reason, future studies should carry out a more fine-grained evaluation of AVH characteristics to better define this particular SZ patients’ subtype^73,74^. In this context, future research might also be oriented to the investigation of the hallucinatory phenomena during the occurrence of the hallucinatory state itself in the acute phase^75,76^, rather than the trait as investigated in the present study. Also, larger datasets would be warranted to corroborate and better interpret our findings.

In agreement with our hypothesis, the fALFF analysis has significantly contributed to increase our knowledge on the association between altered brain functioning and positive symptom (i.e., delusions and hallucinations) severity. It is worth highlighting that the morphometric analysis in these ROIs revealed no significant structural differences among patients and controls that could explain the observed functional differences. This result emphasizes the relevance of functional alterations in explaining the vulnerability to hallucinatory phenomena in SZ patients.

### Supplementary information

Supplementary materials (clean version)
