# Supplementary material for: fMRI fluctuations within the language network are correlated with severity of hallucinatory symptoms in schizophrenia
Source: Schizophrenia (Heidelb). 2023 Oct 30;9(1):75. doi: 10.1038/s41537-023-00401-9 (PMC10616281; doi:10.1038/s41537-023-00401-9)
Supplement: Supplementary file 1 — Supplementary materials (clean version) [file 41537_2023_401_MOESM1_ESM.docx]

Supplementary materials for

fMRI fluctuations within the language network are correlated with severity of hallucinatory symptoms in schizophrenia

Chiara Spironelli, Marco Marino, Dante Mantini, Riccardo Montalti, Alexander R. Craven, Lars Ersland, Alessandro Angrilli, Kenneth Hugdahl

***MRI data processing details***

The structural MRI (sMRI) data preprocessing included Intensity Non-Uniformity (INU) correction and image segmentation, which were performed by using the unified segmentation algorithm implemented in SPM12 (https://www.fil.ion.ucl.ac.uk/spm/), with a regularization parameter equal to 0.0001 and a smoothing parameter equal to 40 mm Full-Width Half-Maximum (FWHM)^[[1]](#footnote-1)^a. fMRI data were preprocessed by means of an automated pipeline developed using SPM12, including spatial alignment to sMRI, motion correction, bias field correction, spatial smoothing (6 mm FWHM), and co-registration to standard space.^1-3^ The fMRI images were analyzed to obtain Language Network (LN) spatial maps from each individual, as well as a LN map at group-level. A Connectivity Analysis was performed, separately for each subject, using spatial Independent Component Analysis (sICA), which was used for decomposing the fMRI data into brain patterns starting from the spatial covariance of the measured signals.^4^ The number of ICs was estimated by using the minimum description length criterion.^5^ Accordingly, 24 to 68 ICs were extracted, depending on the specific fMRI dataset. ICs were calculated using the Fast ICA algorithm proposed by Hyvarinen (1999)^6^, with a deflation approach and hyperbolic tangent non-linearity. This algorithm is implemented in MATLAB (MathWorks, Natick, MA, USA) and can be downloaded at the link <http://research.ics.aalto.fi/ica/fastica/>. For all other analysis, including between-group comparisons and fALFF analyses, Matlab custom scripts were used.^6^ For each IC, a spatial map (that expresses the intensity of the correlation across the voxels of that pattern) and an associated time series (the pattern course over time) were extracted.^7-8^ The spatial map was converted to z-scores by subtracting the average intensity across voxels, and dividing the resulting map by the standard deviation across voxels. An automated template-matching procedure, in which the considered LN-template was derived from previous fMRI study^1^ was used to identify the IC corresponding to LN. The LN-template consists of a group map obtained from single-subject LN maps across 24 healthy subjects by using sICA. In particular, the self-organizing group ICA (sogICA) method by Esposito was used.^6^ In this study, the IC showing the highest spatial correlation with the corresponding template map in Montreal Neurological Institute (MNI) space was identified as the IC of the LN.


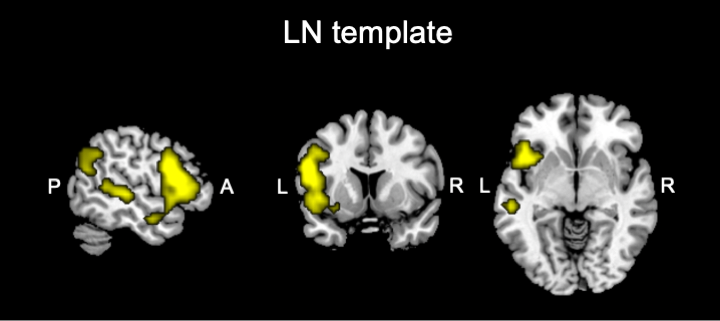


**Figure S1.** The language network (LN) template image used in the present study for the automated template-matching procedure. The P, A, L and R labels stays for posterior, anterior, left and right, respectively.

***References***

1. Mantini, D., Corbetta, M., Romani, G.L., Orban, G.A., Vanduffel, W., 2013. Evolutionarily novel functional networks in the human brain? Journal of Neuroscience 33(8), 3259-3275.
2. Marino, M., Arcara, G., Porcaro, C., Mantini, D., 2019. Hemodynamic correlates of electrophysiological activity in the default mode network. Frontiers in neuroscience 13, 1060.
3. Marino, M., Spironelli, C., Mantini, D., Craven, A.R., Ersland, L., Angrilli, A., Hugdahl, K., 2022. Default mode network alterations underlie auditory verbal hallucinations in schizophrenia. J Psychiatr Res. 155:24-32. doi: 10.1016/j.jpsychires.2022.08.006.
4. McKeown, M.J., Makeig, S., Brown, G.G., Jung, T.P., Kindermann, S.S., Bell, A.J., Sejnowski, T.J., 1998. Analysis of fMRI data by blind separation into independent spatial components. Human brain mapping 6(3), 160-188.
5. Calhoun, V., Adali, T., Pearlson, G., Pekar, J., 2001. A method for making group inferences using independent component analysis of functional MRI data: Exploring the visual system. Neuroimage 13(6), 88-88.
6. Esposito, F., Scarabino, T., Hyvarinen, A., Himberg, J., Formisano, E., Comani, S., Tedeschi, G., Goebel, R., Seifritz, E., Di Salle, F., 2005. Independent component analysis of fMRI group studies by self-organizing clustering. Neuroimage 25(1), 193-205.
7. Mantini, D., Corbetta, M., Perrucci, M.G., Romani, G.L., Del Gratta, C., 2009. Large-scale brain networks account for sustained and transient activity during target detection. Neuroimage 44(1), 265-274.
8. Mantini, D., Perrucci, M.G., Del Gratta, C., Romani, G.L., Corbetta, M., 2007. Electrophysiological signatures of resting state networks in the human brain. Proceedings of the National Academy of Sciences 104(32), 13170-13175.

***sMRI data analyses on ROIs***

**Table S1.** Structural MRI analyses in regions of interest.

| **Contrast** | **Left ROI** | | **Right ROI** | |
| --- | --- | --- | --- | --- |
|  | ***t* value** | ***p* value** | ***t* value** | ***p* value** |
| **HC *vs.* AVH/D–** | 1.473 | *0.150* | 1.417 | *0.166* |
| **HC *vs.* AVH/D+** | 0.187 | *0.852* | -0.340 | *0.735* |
| **AVH/D– *vs.* AVH/D+** | -0.267 | *0.791* | -0.51 | *0.572* |

1. a The term *Full-Width Half-Maximum* is used to describe a measurement of the width of an object in a picture, when that object does not have sharp edges. [↑](#footnote-ref-1)
